# Genetic algorithms for feature selection when classifying severe chronic disorders of consciousness

**DOI:** 10.1371/journal.pone.0219683

**Published:** 2019-07-11

**Authors:** Betty Wutzl, Kenji Leibnitz, Frank Rattay, Martin Kronbichler, Masayuki Murata, Stefan Martin Golaszewski

**Affiliations:** 1 Graduate School of Information Science and Technology, Osaka University, Osaka, Japan; 2 Center for Information and Neural Networks, National Institute of Information and Communications Technology and Osaka University, Osaka, Japan; 3 Institute for Analysis and Scientific Computing, TU Wien, Vienna, Austria; 4 Department of Neurology, Paracelsus Medical University, Salzburg, Austria; 5 Neuroscience Institute, Christian-Doppler Medical Centre, Paracelsus Medical University, Salzburg, Austria; 6 Centre for Cognitive Neuroscience and Department of Psychology, University of Salzburg, Salzburg, Austria; 7 Karl Landsteiner Institute for Neurorehabilitation and Space Neurology, Vienna, Austria; Kochi University of Technology, JAPAN

## Abstract

The diagnosis and prognosis of patients with severe chronic disorders of consciousness are still challenging issues and a high rate of misdiagnosis is evident. Hence, new tools are needed for an accurate diagnosis, which will also have an impact on the prognosis. In recent years, functional Magnetic Resonance Imaging (fMRI) has been gaining more and more importance when diagnosing this patient group. Especially resting state scans, i.e., an examination when the patient does not perform any task in particular, seems to be promising for these patient groups. After preprocessing the resting state fMRI data with a standard pipeline, we extracted the correlation matrices of 132 regions of interest. The aim was to find the regions of interest which contributed most to the distinction between the different patient groups and healthy controls. We performed feature selection using a genetic algorithm and a support vector machine. Moreover, we show by using only those regions of interest for classification that are most often selected by our algorithm, we get a much better performance of the classifier.

## Introduction

In the past decades, emergency treatment as well as intensive care treatment have significantly improved, which has led to more patients surviving severe brain damage. However, many of these patients do not fully recover from their brain damage but remain in some sort of comatose state [1,2]. Hence, the main question that arises during treatment is whether the comatose patient is conscious or not because this has an impact on ethical as well as legal questions. We mainly distinguish between two types of severe chronic disorders of consciousness (scDOC), namely unresponsive wakefulness syndrome (UWS; in the past also called vegetative state (VS) or apallic syndrome), and minimally consciousness state (MCS) [3,4]. Patients suffering from UWS are not aware of themselves nor their environment, but have a day and night rhythm, i.e., they have periods when they sleep and others when they are awake. Some studies found no signs of electrophysiological characteristics of sleep [5,6]. However, newer studies found electrophysiological characteristics of REM (rapid eye movement) as well as non-REM sleep [7,8]. Furthermore, these patients show reflexive responses to external stimuli. On the other hand, besides having such a day and night rhythm, MCS patients also have some kind of awareness of themselves or their environment and hence sometimes (i.e., more often than by chance) respond to external stimuli [4]. Most of the time, the JFK coma recovery scale-revised [9], which is a scale based on behavioral testing, is used for diagnosis and prognosis in daily clinic treatment. When just using simple clinical criteria, a high misdiagnosis rate of up to 43% is evident in this patient group [2,10,11]. Also, when using the JFK coma recovery scale-revised it is important to repeat the examination several times. Not doing so can lead to a misdiagnosis rate of 36% [12]. A new study, which was conducted in Russia in 2017, even found that about 55% of UWS and 80% of MCS patients are initially misdiagnosed [13]. In order to minimize this diagnostic error, our physicians performed a classification with the JFK coma recovery scale-revised and performed multiple repetitions. A misdiagnosis can be devastating for the patients. In general, a wrongful diagnosis will result in an inappropriate level of care for the patient, which could decrease the chance of recovery. Moreover, the diagnosis also has an impact on the prognosis. The prognosis for MCS patients is in general better than for UWS patients [14,15]. In summary, new methods for the diagnosis and prognosis of patients suffering from scDOC are needed.

Recently, neuroimaging technology, especially functional Magnetic Resonance Imaging (fMRI), has been gaining more and more importance for the diagnosis and prognosis of these patients [16–19]. New paradigms and analysis methods have been established in the last few years, utilizing interdisciplinary approaches among which machine learning seems to be a promising tool [20,21]. For example, Höller et al. [22] used different classification methods to divide scDOC patients into the different subgroups according to EEG (electroencephalogram) -features from an imagery paradigm. Moreover, Zheng et al. [23] focused on DTI (diffusion tensor imaging) and machine learning to disentangle disorders of consciousness. They differentiated between patients in VS (UWS), MCS-, and MCS+. As described by Bruno et al. [24], MCS+ patients are those who can express non-functional communication and are able to follow commands whereas MCS- patients are those who can pursue eye following, react to emotional stimuli, and are able to localize painful stimuli. Pugin et al. [25] used resting state fMRI and machine learning to predict the outcome of post-anoxic comatose patients after cardiac arrest. Besides neuroimaging, heart rate variability entropy can also be used for discriminating scDOC patients using machine learning techniques [26]. Especially, classification methods like a support vector machine (SVM) [27] in combination with feature extraction have been successfully applied [28–31]. Most of the papers in that field deal with feature extraction. Such an approach transforms data from the feature space to some target space with dimensionality reduction techniques, e.g., principal component analysis (PCA), see for example [29]. This strategy is difficult to interpret if we want to identify the most important features in the original feature space.

On the other hand, there is also the technique of feature selection which refers to eliminating those features in the original feature space that do not contribute to the classification. In this paper, we combine a genetic algorithm (GA) [32] and SVM for selecting the most important features when distinguishing between healthy controls and scDOC patients as well as between the two patient groups. The approach to combine GA and SVM is well-established, see e.g. [33–35]. When considering classification of fMRI data, we face the problem of having a very high dimensional input space. Irrelevant features can increase the run time as well as the cost of the system while giving poorer results. Hence, we aim at finding the part of the input data that contributes most to the classification. On the one hand, we want to find the smallest possible number of features but, on the other hand, also want to retain the best results from the classification. Such a search is in general referred to as feature selection. One popular approach is recursive feature elimination whereby features are removed from the feature set and changes in classification accuracy can be used to assess the relative importance of any feature. Most algorithms use Pearson’s cross-correlations of any pair of ROI time series as features, while we aim for individual ROIs as features. Hence, the standard algorithms, like recursive feature elimination, are not directly applicable. In the python library scikit-learn [36], there are implementations of univariate feature selection (e.g. SelectKBest), which look at the contribution of each feature independently, or multivariate methods, such as Recursive Feature Elimination [37]. All these methods find the most important features, i.e., entries of the correlation matrix, for the classification. However, this is not what we want here as we try to find the most important ROIs and not the correlation between ROIs. In all of the previously mentioned approaches, features would be selected as pairs of ROIs. However, since we are interested in the impact of individual ROIs as features, directly applying feature selection to the correlation matrix is not appropriate.

The filter method or the wrapper method are in general used for feature selection. In the filter approach the features are preselected during the preprocessing procedure with regards to a predefined relevance measure which is independent of the performance of the learning algorithm. A predefined relevance measure can be, for example, a One-Way-Anova. The One-Way-Anova compares the given dataset and returns single p-values which are used to rank the importance of the features of which the most important ones are used for further preprocessing [38]. Such a filter method is robust to overfitting and computationally effective. On the other hand, wrapper methods use a given subset of features as input for the classifier and estimate this subset’s performance as criterion for its relevance. This is done for each of these subsets. The wrapper method is in general more powerful but, on the other hand, it is also computationally more demanding and often faces the problem of overfitting. A newly proposed method is the embedded method during which the feature selection is directly combined with the learning algorithm [39–42]. In this paper we will use the wrapper approach, i.e., we use the results of the SVM as a measure of the subset of features selected by the GA. Up to now this methodology has not been used to distinguish between the two different types of scDOC and healthy controls.

## Material and methods

### 2.1. Experiment

Before starting the experiments, we obtained the approval of the Ethics Commission Salzburg for this study (Ethikkommission Land Salzburg, number 415-E/952). Written consent was obtained from all participants or legal guardians of the scDOC patients. The measurements were performed over a course of several years. This is the reason why two different scanners were used for this experiment, namely, a 3 Tesla Philips Achieva (Philips, Amsterdam, Netherlands) and a 3 Tesla Siemens Tim Trio (Siemens, Erlangen, Germany). We measured 30 healthy subjects (12 with the Philips scanner) and 58 patients (16 with the Philips scanner). The patients had different etiologies, sex, and ages (see S1 Table for details). The healthy controls had a mean age of 44.17 years (range 20 to 79, standard deviation 19.45) and 22 of them were male. All healthy controls had no history of previous neurological or psychiatric disorders.

We acquired T2*-weighted echo-planar sequence images in axial plane with the 3 T Philips scanner. The parameters were: repetition time 2.2 s, echo time 45 ms, 25 slices with a slice thickness of 4.5 mm (inter-slice gap 0.5 mm), flip angle 90 degrees, matrix size 64x64, and field of view 210 mm^2^. The parameters of the 3 T Siemens scanner for the T2*-weighted echo-planar sequence images in the axial plane were as follows: repetition time 2.25 s, echo time 30 ms, 36 slices with a slice thickness of 3 mm (no interslice gap), flip angle 70 degrees, matrix size 92x92 and field of view of 192 mm^2^. Furthermore, we acquired a T1-weighted MPRAGE sequence for each healthy subject and each patient.

### 2.2. Extracting ROI time series and correlation matrix

For preprocessing we used the CONN functional connectivity toolbox [43] which is based on Statistical Parametric Mapping (SPM, https://www.fil.ion.ucl.ac.uk/spm/) and MATLAB (The MathWorks, Inc., Natick, Massachusetts, United States). The default pipeline without smoothing was applied. This pipeline begins with realignment (rigid-body transformation with six parameters) to compensate for head motion. The next step is unwarping, which adjusts for movement-related artifacts. Then the data are centered and corrected for slice-timing, i.e., compensation for the fact that not all slices have been acquired instantaneously but with a time delay of a few seconds (depending on the repetition time), for further details see [44]. The next step in the pipeline is outlier detection which is performed with the Artifact Detection Toolbox (ART). We used the 97^th^ percentile as threshold. Next follows direct segmentation and normalization so that we could compare the results of the different subjects. These steps are all done for the functional data. Centering, direct segmentation, and normalization are also performed for the structural data. We included these steps to find out the volume of the different brain matters which were later used as confounds (see Section 2.3). After this pipeline, we applied denoising. During each of these steps we conducted quality control and also checked the data visually. We had to exclude 9 subjects due to various reasons, e.g., movement artifacts. Hence, a total number of 30 healthy subjects, 29 UWS, and 20 MCS could be used for further analysis (see S1 Table for which subjects have been included). With these remaining subjects we performed a first level region of interest (ROI) to ROI analysis using the CONN atlas consisting of 132 ROIs, including the cortical and subcortical areas of the FSL Harvard-Oxford atlas and the cerebellar areas of the AAL atlas. We obtained for each subject the Fisher Z-transformed full correlation matrices of the Blood-Oxygenation Level Dependent (BOLD) time series of all ROIs as a result.

### 2.3. Scanner differences

The fact that we used two different scanners for our experiments, could have introduced a bias to our data. That is why we first performed a connectivity analysis of the data using the scanner as dummy variables, i.e., subjects scanned with the Philips scanner got a 0 and subjects scanned with the Siemens scanner got a 1. These variables alongside with other confounds, namely valid scans, invalid scans, maximum motion, mean motion, maximum global signal, mean global signal, grey matter volume, grey matter volume eroded, white matter volume, white matter volume eroded, cerebrospinal fluid volume, cerebrospinal fluid volume eroded, global correlation for rest, patients, healthy controls, UWS patients, and MCS patients were used in the model. We included all these possible confounds to make sure that the gained results were just due to the scanner differences and not due to any of the above mentioned confounds. Then we performed a second level ROI-to-ROI analysis with CONN to find out which connections were stronger or weaker when comparing the Philips to the Siemens scanner.

### 2.4. GA-based feature selection

This part was performed using python version 2.7 (Python Software Foundation, https://www.python.org https://www.python.org/).

While all of the 132 ROIs, obtained from the CONN preprocessing steps, could be regarded as possible features, not all of them contribute necessarily equally to the classification between the four subject classes of interest, namely healthy controls, patients, MCS patients, and UWS patients. Therefore, we were interested in determining which features, i.e., ROIs, contributed most to the correct classification of the subject classes.

**Fig 1:**
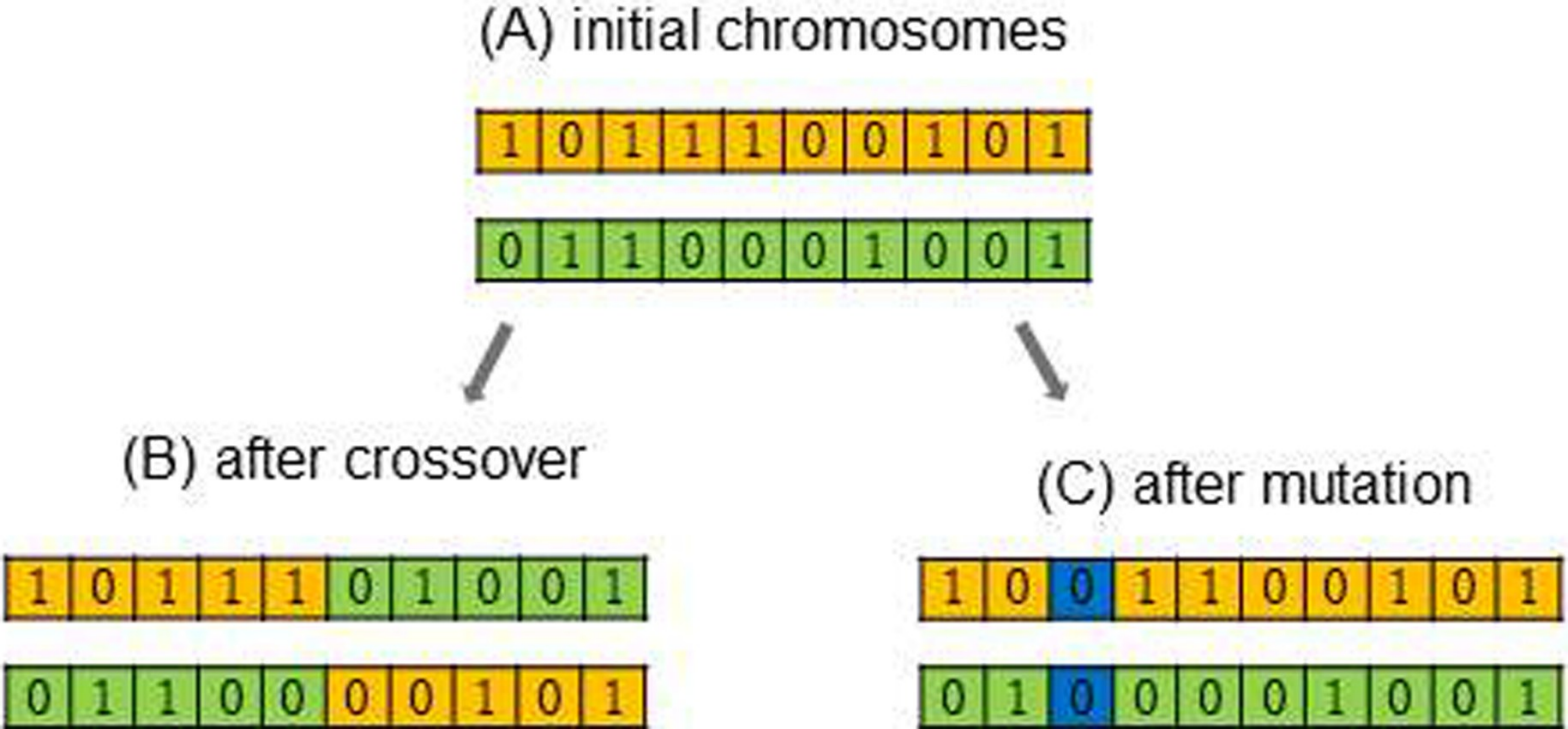
Graphical representation of crossover and mutation in GA. (A) shows two parent chromosomes. The second row shows the children’s chromosomes after crossover (B) and after mutation (C).

In our approach, we propose applying the meta-heuristic of GAs to determine the minimal subset of features, i.e., ROIs, for which the different classes are best distinguished during classification. In general, a GA mimics the evolutionary dynamics of a finite population of individuals (phenotype), where each individual represents one candidate solution and consists of a vector of chromosomes (genotype), which is a Boolean vector indicating if a feature should be included or not. The GA is iteratively performed over several generations and reproduction is performed to obtain individuals that are a best fit for a certain environment. Fitness is the key measure to quantify how well an individual is suited to the environment and only individuals with high fitness will survive over time. Reproduction is mimicked by performing crossovers and mutations. In crossover, parts of two parents’ chromosomes are swapped to form the chromosomes of their children. While this contributes to a large step in the search for a better solution, a mutation only modifies individual chromosomes and contributes to a local search for a better solution. See Fig 1 for a graphical representation of crossover and mutation.

**Fig 2:**
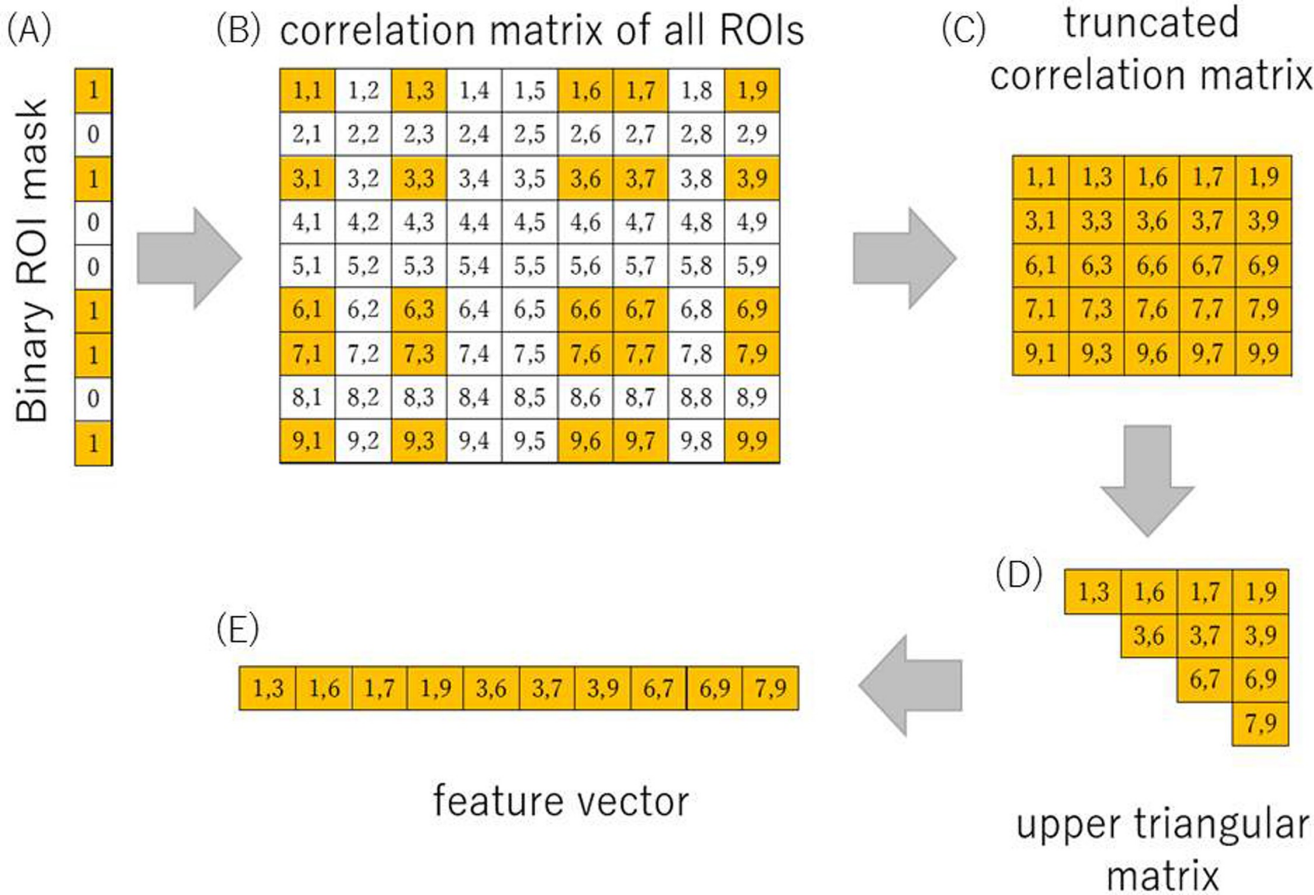
Representation of the feature selection algorithm. The binary vector (A) is applied to the correlation matrix (B) (for graphical representation this is only a 9x9 matrix—in our actual analysis this was a 132x132 matrix), selecting only those combination of ROIs which both have a “1” in the binary mask. This results in a smaller truncated correlation matrix (C). Due to the symmetry of this matrix, we just use the upper triangular matrix for further calculation. This triangular matrix (D) is then flattened into a feature vector (E). Each integer tuple shown in the matrix represents its row and column indices, i.e., i,j corresponds to row i, column j.

Now we turn to the specific parameters we use in our approach. Instead of using all features for classification, we look at the performance when we select a subset of ROI features. We define a mask of binary values (genotypes), indicating if an ROI is included (value 1) in the classification or not (value 0). A population of phenotypes is initialized where each ROI is randomly set to 1 with a probability of 0.25 and to 0 with probability 0.75. We then perform a GA on this population with mutations and crossovers over 100 generations. It is worth mentioning that any result found by a GA can always improve with more generations but can never become worse. Hence, when the algorithm finds a good solution it will be kept until a better one is found. For the fitness calculation of one individual, we apply the chromosome as a binary filter to the rows and columns of the correlation matrix, indicating whether a feature should be included or not, see Fig 2. This results in a smaller matrix (Fig 2C), of which we extract the upper triangular matrix (the matrix is symmetric) which is then flattened to a feature vector. The target value of this feature vector indicates the class it belongs to. We use SVM to evaluate how well the reduced features help distinguishing between the classes.

While the performance of a classifier is often evaluated in terms of accuracy, this metric frequently suffers in quality when dealing with imbalanced datasets, which we have in our study. We therefore selected as fitness the area under curve (AUC) value of the precision-recall plot because it is known to be less dependent on imbalanced classes [45]. The metrics precision and recall are defined as follows

$$precision=\frac{TP}{TP+FP},\;recall=\frac{TP}{TP+FN}$$ (1)

with *TP* being the true positives, *FP* the false positives and *FN* the false negative [45], [46]. Precision is also referred to as *positive prediction rate* and recall is also called *true positive rate* or *sensitivity*.

The other parameters for the GA were selected as follows. The population size was 1000, the number of generations was 100, and the number of elites was 5, i.e., 5 individuals were kept without modifications for the next round. The mutation probability was 0.1. Since we are looking for solutions with only a few features active, we modified mutations to only take on transitions from 1 to 0. Since such an approach made our method get easier stuck in local minima, while searching for the best solution, we repeated the overall GA 1000 times to consider a wide range of runs on our search for the best locally minimal value. Since SVM only permits the comparison of two classes at a time, we tested all four possible combinations as four separate runs: (R1) healthy versus patients (i.e. MCS and UWS together), (R2) healthy versus MCS, (R3) healthy versus UWS, and (R4) MCS versus UWS.

### 2.5. Overall algorithm

**Fig 3:**
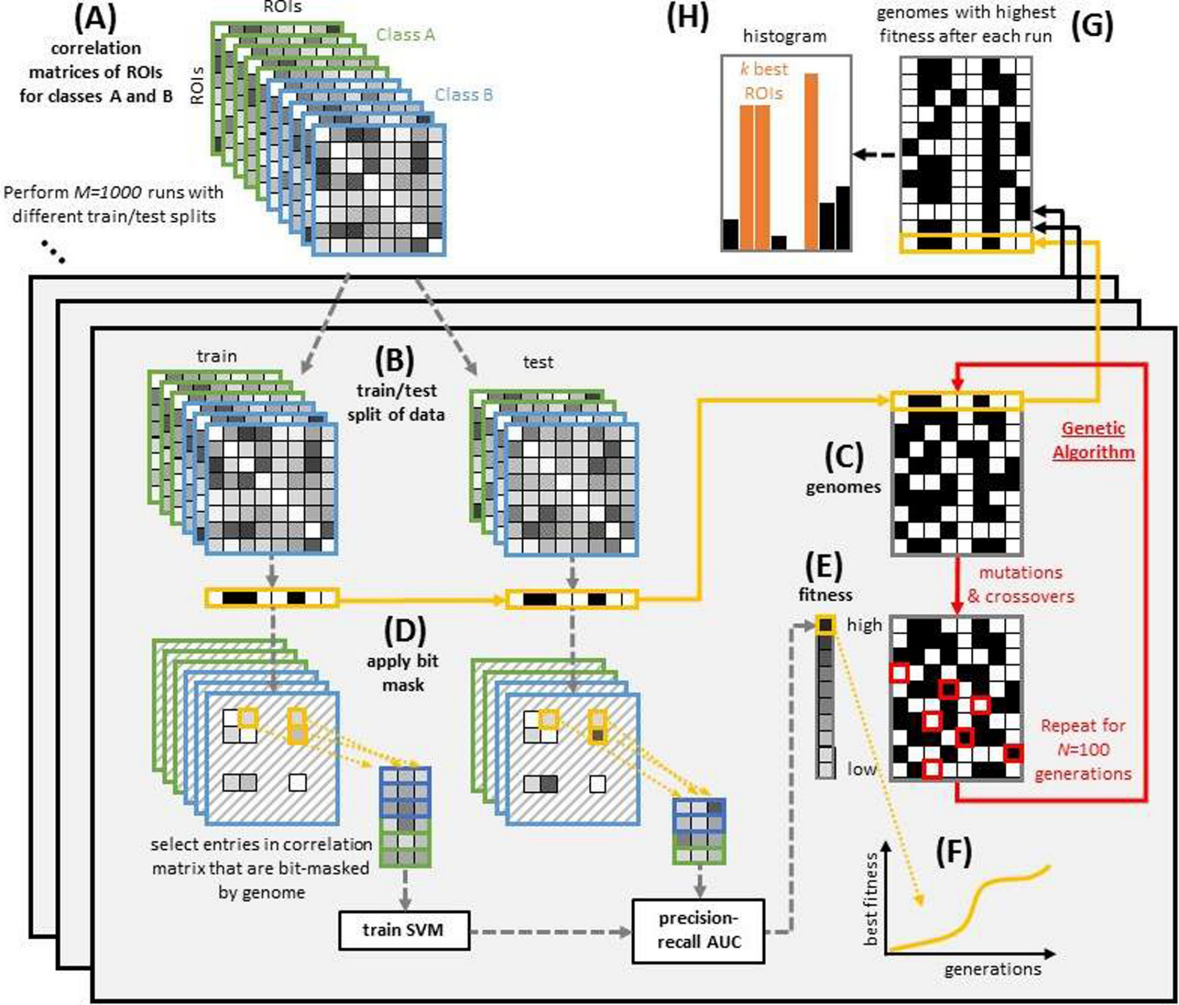
Schematic view of the overall algorithm. We start with the correlation matrices (A). The classes A and B stand for any two classes of healthy controls, patients, UWS, or MCS. The correlation matrices are split into a train-test set (B). The training set is first used for the GA. We find a binary mask as the best solution of the GA (C). This binary mask is then applied to the correlation matrices (D) (see also Fig 2). Then the fitness is calculated from the test set for each genome (E) and a fitness function is plotted (F). This plot shows the fitness of the best genome of each generation. After the GA is repeated 100 times, the best genome is saved which results in 1000 best binary ROI masks (G). In order to find the best ROIs, we plot histograms of these results (H). These best results are used for further calculations.

Fig 3 gives an overview of the entire algorithm. We start with the correlation matrices of two classes (class A and class B) as any among (R1)-(R4). All samples from classes A and B are first merged, while we leave out the samples not belonging to either of the classes A or B. For each of the 1000 repetitions of the GA we separate the merged classes into a training set and a test set. The training set is used for the GA to determine the best ROI mask pattern. Then the upper triangular matrices of these symmetric and truncated matrices are unfolded into 1-dimensional feature vectors which are input to the SVM (see Fig 2). After training the SVM, we use the test set for evaluation. Thus, the same ROIs as for the training set are masked out of the correlation matrices for the test set. After that, the area under the curve for the precision-recall plot is calculated and the fitness is determined. This is done for 100 generations for the same train-test split and we save the best solution of the GA. The train-test split is calculated for every run of the GA, i.e., it is different for every run. Each of the 1000 runs yields as solution a Boolean vector over all ROIs indicating which ROIs should be selected. A histogram over all of these result vectors illustrates how often one specific ROI has been included in the result over all runs.

**Fig 4:**
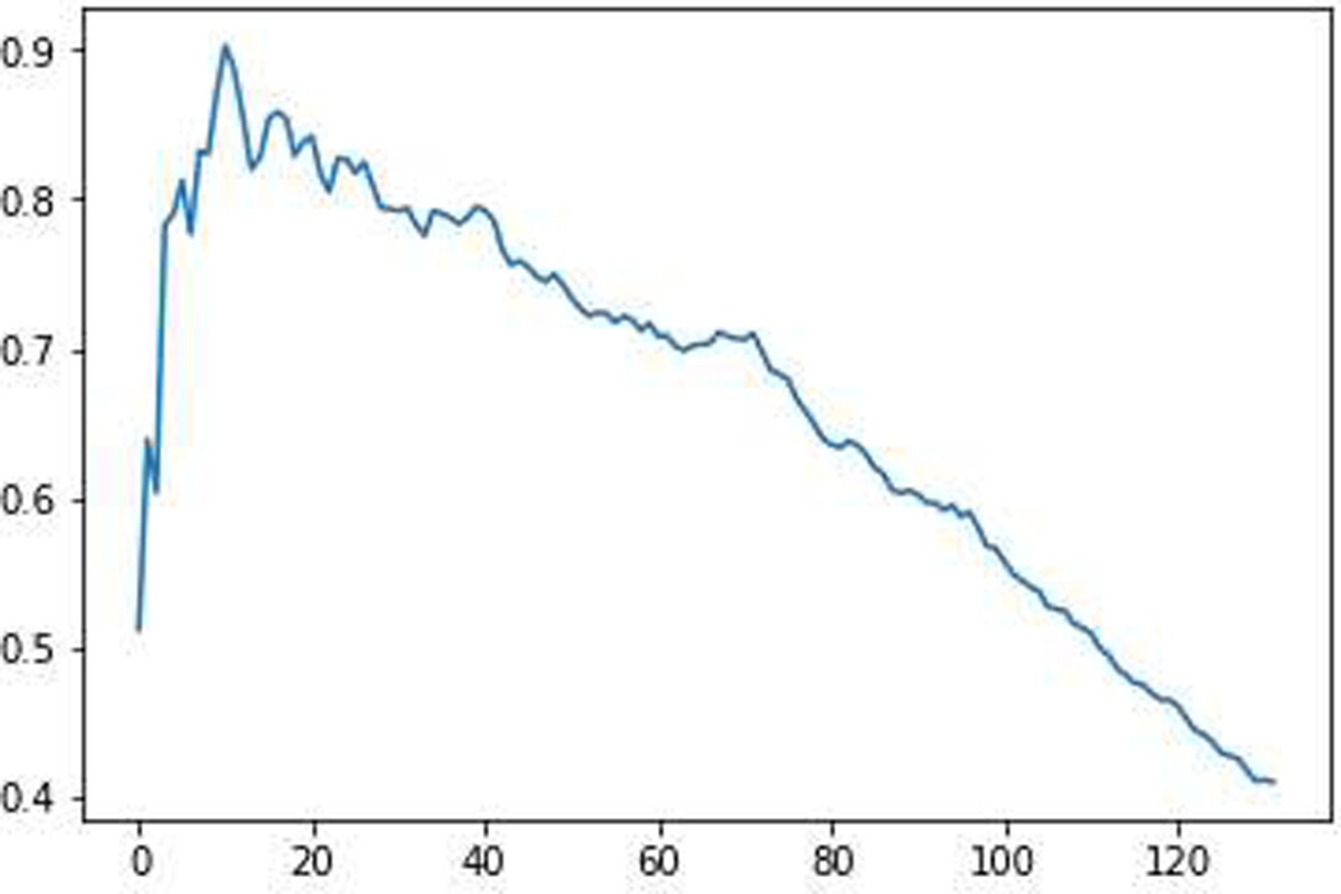
Averaged AUC values over 1000 repetitions when using a certain number of ROIs for differentiating healthy subjects from UWS patients. The maximum is a number of 10 ROIs. The x-axis gives the number of ROIs and the y-axis gives the value of the averaged AUC.

First, we calculated the histogram of how often the ROIs were chosen to be important by the GA. We sorted these ROIs in accordance with their frequency. Then we calculated the AUC of the precision-recall curve when choosing the n best ROIs. During this algorithm a train-test split with a ratio of 0.33 was performed in every run and the whole procedure was repeated 1000 times, and then the AUC was averaged over all runs. These averaged values were plotted in a diagram (see Fig 4 for an example) and the maximum was found.

Moreover, we performed a permutation test to rule out that using a smaller number of ROIs yielded better results because of smaller correlation matrices as input. This permutation test was done by randomly choosing a certain number of n ROIs 100000 times and calculating the AUC of the precision-recall curve. Again, there was a train-test split in each iteration with a percentage of 0.33. This split was different for each iteration.

## Results

We found that no connections differ between the different scanner types with a p-value of 0.05 p false discovery rate (analysis level correction). Hence, the different scanner types did not introduce any bias to our study.

**Fig 5:**
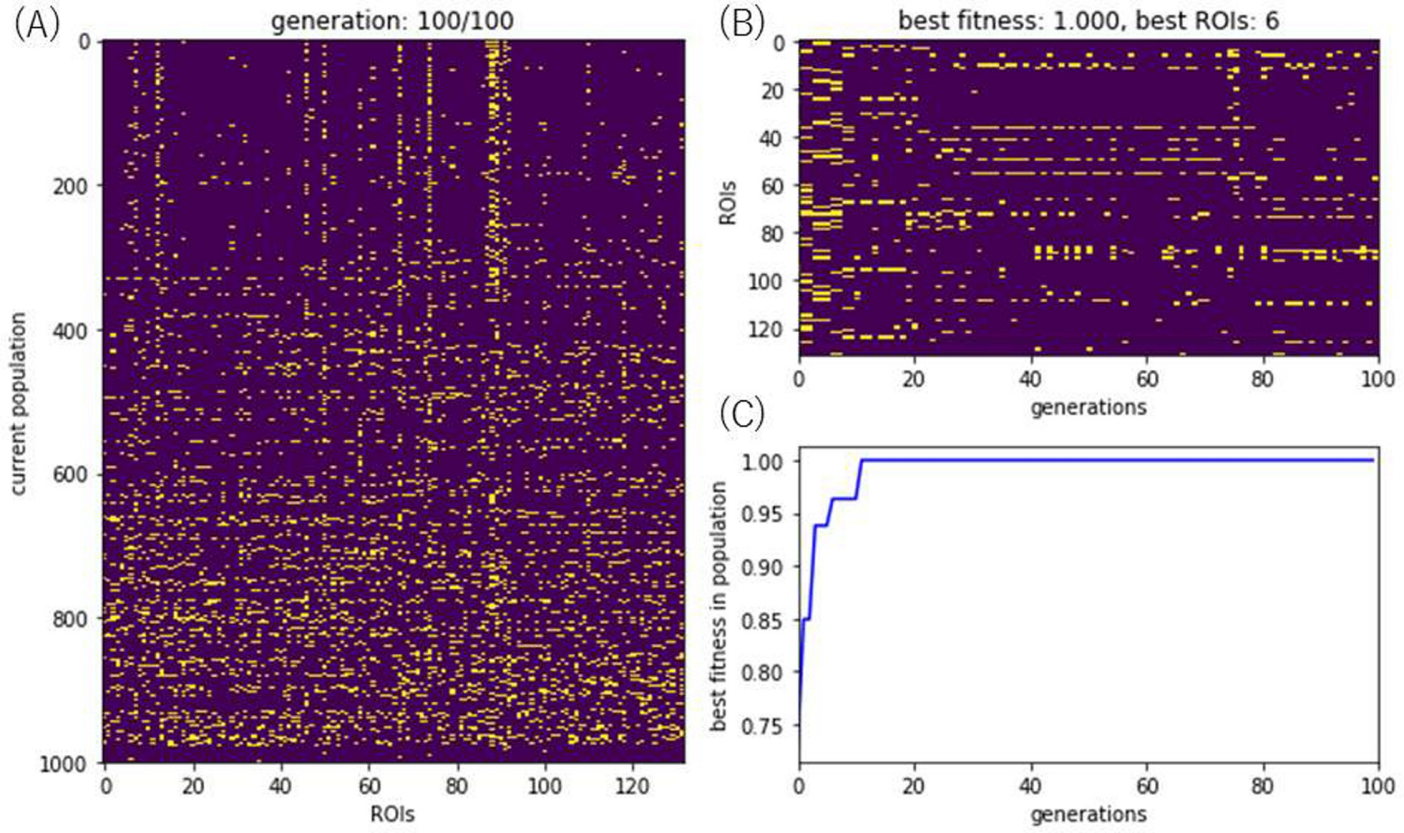
Example of a single run of the GA for feature selection. The ROIs selected as features are shown in yellow in subfigure (A) (corresponds to Fig 3C). This subfigure (A) shows the entire current population at the 100th generation where each row represents one individual and rows are sorted by fitness in descending order. The subfigure (B) shows the chosen ROIs of the solution with highest fitness for each generation (corresponds to Fig 3G), ending with the one which just has 6 ROIs, i.e. 6, 58, 67, 88, 89, and 91. The subfigure (C) represents the best fitness of the population (corresponds to Fig 3F) starting at around 0.85 and ending at a fitness of 1 which represents the AUC of the precision and recall curve.

Fig 5 shows a representation of a single run of the GA which found as a best solution the six ROIs with the numbers 6, 58, 67, 88, 89, and 91 namely *superior frontal gyrus left*, *cuneal cortex right*, *lingual gyrus left*, *supracalcarine cortex right*, *supracalcarine cortex left* and *occipital pole left*. These selected ROIs resulted in a fitness of one, i.e., the area under the precision and recall curve was one. When comparing the fitness to that of the initial population with random ROIs, its value rose from 0.85 to 1. However, this is just one example of a single run of the GA. Nevertheless, the results of the other runs are quite similar.

**Fig 6:**
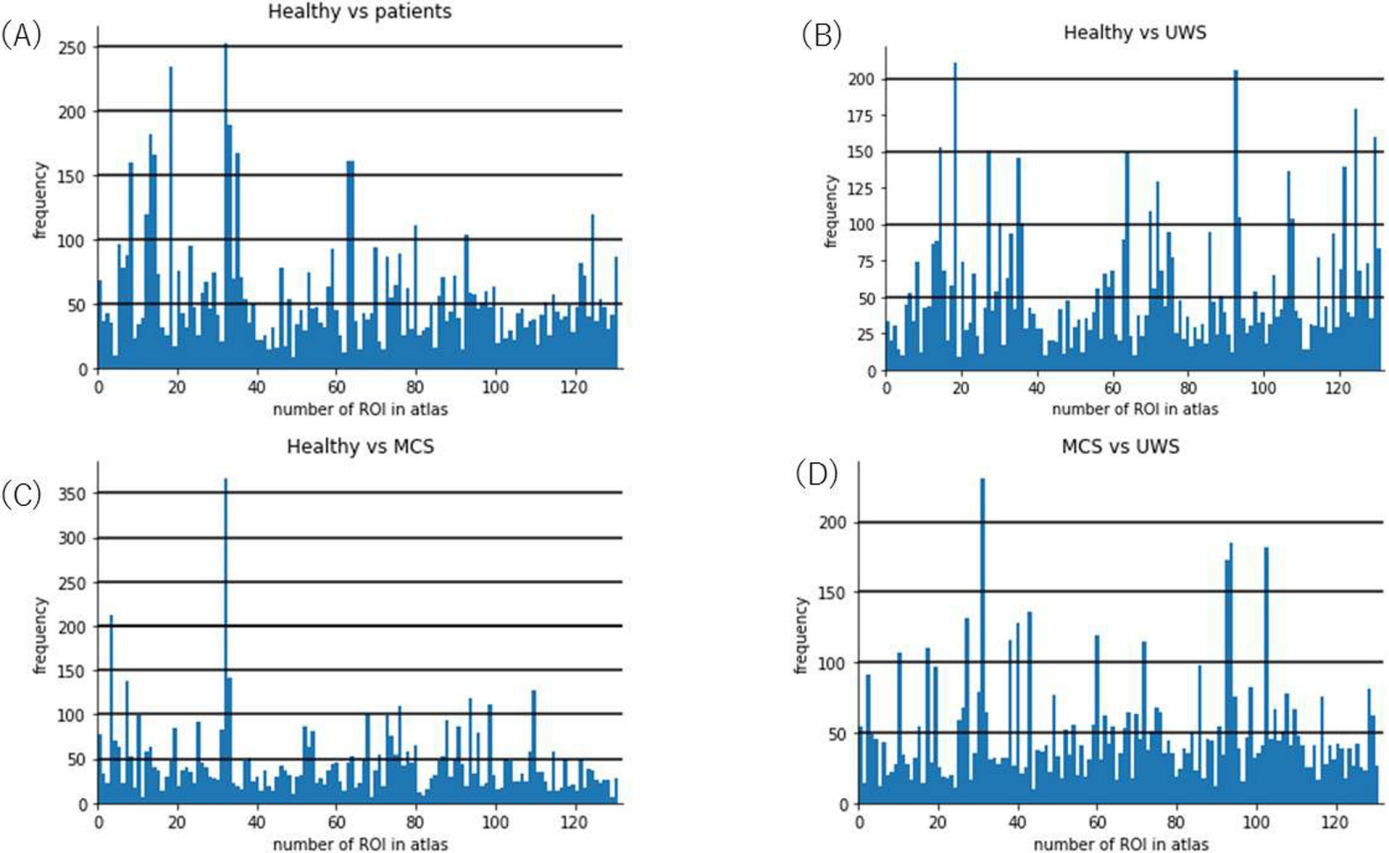
Histograms of the different GA results (corresponds to Fig 3H) when comparing the four different combinations (healthy controls versus patients, healthy controls versus MCS, healthy controls versus UWS, and MCS versus UWS). The x-axis shows the ID of the ROI in the CONN atlas and the y-axis shows its frequency, i.e., how often the GA chose this ROI to be important.

We found the ROIs that were most often chosen by the GA to be important for the classification with SVM. Selecting these k-best ROIs is done by repeatedly (1000 times) calculating the AUC of the precision-recall curve of the SVM at a train-test split percentage of 0.33 and finding the number of ROIs that yielded the highest value of the AUC. A different number of ROIs is important for each combination. Hence, the comparison between UWS and MCS patients gives 22 ROIs as the maximum (AUC = 0.9397), healthy versus patients gives 17 ROIs (AUC = 0.9015), healthy versus UWS gives 10 ROIs (AUC = 0.9026), and healthy versus MCS shows a maximum of the AUC of the precision-recall curve for 6 ROIs (AUC = 0.8392). When analyzing healthy subjects versus patients the most important ROI was the *postcentral gyrus right*, which was also the most important ROI for distinguishing healthy subjects from MCS patients. The second and third most important ROI for distinguishing healthy subjects from patients were the *superior temporal gyrus posterior division right* and the *postcentral gyrus left*. When considering healthy subjects versus MCS, we find as second most important the *insular cortex left* and as third most important again the *postcentral gyrus left*. The third group of healthy versus UWS has as the most important ROI the *superior temporal gyrus posterior division right* which is also the second most important one when distinguishing healthy from patients. The second most important ROI is the *caudate right* and the third is the *vermis 3* which also shows importance for distinguishing healthy subjects versus patients. The last group, when distinguishing UWS versus MCS, yields quite different ROIs, namely *inferior temporal gyrus*, *temporooccipital part left*, *caudate left*, and *accumbens right*. Besides this, one finds that *caudate right*, *inferior temporal gyrus anterior division left*, and *temporal occipital fusiform cortex left* are important for distinguishing healthy controls from UWS as well as MCS from UWS. The most important ROIs can be found in Fig 6 which shows histograms of how often an ROI was chosen by the GA to be in the best fit solution for all 1000 runs. Each histogram shows one of our four combinations. What can be seen, when considering the plot for healthy versus MCS, is that the *postcentral gyrus right* seems to be of special importance because its selection frequency is a lot higher than that of the other ROIs. When considering the other three histograms one also finds pronounced ROIs but those are not as strong as this one. A summary of the most notable ROIs and their selection frequency can be found in Table 1. Last but not least, we confirmed that the performance of the classifier was higher when we just selected these eleven ROIs instead of using all 132 ROIs. The results are shown in Table 2. We find that all the accuracies and AUC of the precision-recall curve increased. The results of the permutation test showed, that all results given in Table 2 are statistically significant with a p-value of 0.00001 uncorrected.

**Table 1:** Most frequent ROIs when comparing healthy controls and patients, healthy controls and MCS patients, healthy controls and UWS, or MCS and UWS patients. The columns are the name of the ROI, its ID in the CONN atlas, and its selection frequency (how often this ROI was chosen by the GA as one of the most important ones). Bold ROIs show up in more than one comparison.

	Healthy vs patients			MCS vs UWS		
	ROI	Nr.	count	ROI	Nr.	Count
1	**postcentral gyrus right**	**33**	253	inferior temporal gyrus, temporooccipital part left	32	231
2	**superior temporal gyrus, posterior division right**	**19**	234	**caudate left**	**95**	185
3	**postcentral gyrus left**	**34**	189	accumbens right	104	182
4	precentral gyrus left	14	182	**caudate right**	**94**	173
5	**superior parietal lobule left**	**36**	167	lateral occipital cortex, superior division left	44	136
6	**temporal pole right**	**15**	166	**inferior temporal gyrus, anterior division left**	**28**	131
7	**parahippocampal gyrus, posterior division left**	**65**	161	angular gyrus right	41	128
8	parahippocampal gyrus, posterior division right	64	161	frontal orbital cortex left	61	119
9	inferior frontal gyrus, pars triangularis right	9	160	supramarginal gyrus, posterior division right	39	116
10	**vermis 3**	**126**	119	temporal occipital fusiform cortex left	73	115
11	precentral gyrus right	13	119	superior temporal gyrus, anterior division left	18	110
12	parietal operculum cortex left	81	111	inferior frontal gyrus, pars opercularis right	11	107
13	**caudate right**	**94**	104	planum temporale left	87	98
14	superior frontal gyrus left	6	96	superior temporal gyrus, posterior division left	20	97
15	middle temporal gyrus, posterior division left	24	95	insular cortex right	3	91
16	temporal fusiform cortex, posterior division left	71	94	hippocampus right	100	82
17	inferior frontal gyrus, pars triangularis right	10	93	vermis 8	130	81
18				inferior temporal gyrus, temporooccipital part right	31	79
19				cerebelum crus2 left	109	78
20				juxtapositional lobule cortex -formerly supplementary motor cortex- right	50	77
21				cerebelum 7b right	118	76
22				putamen right	96	76
	Healthy vs UWS			Healthy vs MCS		
	ROI	Nr.	count	ROI	Nr.	count
1	**superior temporal gyrus, posterior division right**	**19**	211	**postcentral gyrus right**	**33**	367
2	**caudate right**	**94**	102	insular cortex left	4	212
3	**vermis 3**	**126**	179	**postcentral gyrus left**	**34**	142
4	vermis 9	131	160	middle frontal gyrus left	8	138
5	**temporal pole right**	**15**	153	cerebelum 3 left	111	128
6	**inferior temporal gyrus, anterior division left**	**28**	151	**caudate left**	**95**	118
7	**parahippocampal gyrus, posterior division left**	**65**	150			
8	**superior parietal lobule left**	**36**	145			
9	cerebelum 10 left	123	139			
10	cerebelum crus1 right	108	136			

**Table 2:** Table of averaged results over 100 train-test splits with a ratio of 0.33. The AUC of the precision and recall curve is shown (AUC with feature selection) for training and testing with the most important ROIs and with all ROIs (AUC without feature selection).

	Healthy vs patient	Healthy vs MCS	Healthy vs UWS	MCS vs UWS
**AUC** with feature selection	0.8239	0.7950	0.8871	0.8628
**AUC** without feature selection	0.5018	0.3886	0.4116	0.6159

## Discussion

Our first finding is that the proposed algorithm works well and that it is applicable to patients with scDOC. The fact that some ROIs occur more often for different comparisons shows that the algorithm is consistent. Choosing the approach of using feature selection directly gave us the ROIs that are most important for the classification.

Using only the most important features for SVM gives us statistically better results than using all of the ROIs (permutation test with p = 0.00001 uncorrected). This shows that it is indeed better to use only the determined fraction of ROIs and that there are a lot of ROIs that add noise and do not contribute to the real classification.

According to our findings the *postcentral gyrus right* seems to play an important role in MCS patients. Its frequency is highest when looking at the comparison of healthy controls versus patients and also healthy controls versus MCS. In the second case it is especially pronounced. On the other hand, turning to UWS patients we find that the *superior temporal gyrus*, *posterior division right* is important because it has the highest frequency when comparing healthy controls and UWS patients and also is the second most important feature for the comparison of healthy controls and patients. The distinction of MCS versus UWS shows quite different results and only shows three ROIs (when considering the most pronounced 22 ones) that are also important in other comparisons, namely, healthy subjects versus UWS patients and just one, i.e., *caudate right*, that is also important when comparing healthy to patients.

The described approach seems to be a promising tool in the field of neuroscience. Nevertheless, it has its limitations. One of the drawbacks is that the brainstem and the cerebellar anatomical regions are missing in the important ROIs. Brainstem lesions seem to play an important role in scDOC [47–49]. Hannawi et al. [50] performed a meta-analysis showing that the activity in the anatomical structures linked to the default-mode network, midline cortical, and subcortical sites are decreased in patients with scDOC. They also mention studies using PET, SPECT, and a combination of both, which are also often used tools when diagnosing these patients. Nevertheless, we did not use them because they are invasive methods. Moreover, we have to mention that this study only focused on resting state networks. We used resting state scans because it is the easiest measurement for this patient group. However, studies using task fMRI for this patient group have obtained good results as well, see for example [51–53]. Hence, it may be wise to combine resting state analyses and task-based fMRI. Especially including task-based fMRI in the diagnosis could lead to improvements and, thus, make the whole algorithm, where the diagnosis is used as ground truth, more accurate. Moreover, we have to keep in mind the circular logic when testing new methods using old methods as criteria because the aim is to improve the old methods by means of the new ones. We see this study as a first step in developing this new method and as this, we applied the diagnostic criteria even if it is not that reliable. The outcome criterion might substantially increase the reliability, but a study based upon the outcome criterion would be considerably more expensive as regards time, money and size of patient groups.

Furthermore, it would be interesting to also analyze the subtypes of MCS, i.e. MCS+ and MCS-. Further, important patient groups which would be suitable for such a distinction are locked-in syndrome (LIS) and locked-in plus syndrome (LIPS) patients. The former describes patients that are fully aware of themselves and their environment but just have problems moving their bodies [54]. LIPS patients describe patients who are in LIS, hence, cannot (or just partly) move their bodies but also have some form of scDOC [55].

In the future the combination of GA and SVM shall also be applied to other neurological diseases, especially dementia, where specially supratentorial features can distinguish different pathological entities, e.g., in early stages of dementia or other neurogenerative diseases, multiple sclerosis, brain tumor, or cognitive and psychiatric disorders.

## Supporting information

S1 Table Overview over all the patients included in the study. P stands for the 3T Philips scanner and S for the 3T Siemens scanner. Y stands for patients that have been included and N for patients that have not been included into the analysis.
(DOCX)
