# Supplementary material for: Genetic algorithms for feature selection when classifying severe chronic disorders of consciousness
Source: PLoS One. 2019 Jul 11;14(7):e0219683. doi: 10.1371/journal.pone.0219683 (PMC6622536; doi:10.1371/journal.pone.0219683)
Supplement: S1 Table — P stands for the 3T Philips scanner and S for the 3T Siemens scanner. Y stands for patients that have been included and N for patients that have not been included into the analysis. (DOCX) [file pone.0219683.s001.docx]

**Supporting information:**

**S1 Table:** Overview over all the patients included in the study. P stands for the 3T Philips scanner and S for the 3T Siemens scanner. Y stands for patients that have been included and N for patients that have not been included into the analysis.

| Subject | Age [yrs] | sex | Time since onset [d] | Etiology | scanner | included in study |
| --- | --- | --- | --- | --- | --- | --- |
| UWS1 | 45 | F | 64 | traumatic brain injury | P | N |
| UWS2 | 45 | M | 635 | cardiopulmonary resuscitation | P | Y |
| UWS3 | 50 | M | 204 | cardiopulmonary resuscitation | P | N |
| UWS4 | 69 | M | 58 | cardiopulmonary resuscitation | P | Y |
| UWS5 | 39 | F | 73 | respiratory failure | P | Y |
| UWS6 | 47 | M | 65 | cardiopulmonary resuscitation | P | Y |
| UWS7 | 45 | M | 182 | traumatic brain injury | P | Y |
| UWS8 | 29 | F | 104 | basilaris thrombosis | P | N |
| UWS9 | 78 | M | 39 | cardiopulmonary resuscitation | P | Y |
| UWS10 | 47 | F | 51 | Multiple ischemic infarct | P | Y |
| UWS11 | 63 | M | 16 | subarachnoidal haemorrhage | P | Y |
| UWS12 | 51 | M | 30 | cardiopulmonary resuscitation | P | Y |
| UWS13 | 50 | M | 165 | Ischemic brainstem infarct | S | Y |
| UWS14 | 51 | F | 1474 | cardiopulmonary resuscitation | S | Y |
| UWS15 | 38 | F | 78 | subarashnoidal haemorrhage | S | Y |
| UWS16 | 55 | F | 121 | cardiopulmonary resuscitation | S | Y |
| UWS17 | 61 | M | 116 | traumatic brain injury | S | Y |
| UWS18 | 26 | M | 124 | traumatic brain injury | S | N |
| UWS19 | 55 | M | 47 | metabolic encephalopathy | S | N |
| UWS20 | 54 | M | 70 | cardiopulmonary resuscitation | S | Y |
| UWS21 | 59 | M | 20 | traumatic brain injury | S | Y |
| UWS22 | 55 | M | 182 | cardiopulmonary resuscitation | S | Y |
| UWS23 | 73 | M | 59 | traumatic brain injury | S | Y |
| UWS24 | 43 | M | 68 | cardiopulmonary resuscitation | S | Y |
| UWS25 | 52 | M | 611 | venous sinus thrombosis | S | Y |
| UWS26 | 82 | M | 27 | cardiopulmonary resuscitation | S | Y |
| UWS27 | 68 | M | 43 | cardiopulmonary resuscitation | S | Y |
| UWS28 | 30 | M | 502 | traumatic brain injury | S | Y |
| UWS29 | 18 | M | 569 | hyperthermia | S | Y |
| UWS30 | 18 | M | 41 | encephalopathy | S | Y |
| UWS31 | 17 | M | 94 | encephalopathy | S | Y |
| UWS32 | 33 | M | 80 | encephalopathy | S | Y |
| UWS33 | 48 | F | missing | intracerebral haemorrhage | S | Y |
| UWS34 | 66 | M | 60 | traumatic brain injury | S | Y |
| MCS1 | 40 | M | 1887 | traumatic brain injury | S | Y |
| MCS2 | 54 | F | 640 | subarachnoidal haemorrhage | P | Y |
| MCS3 | 51 | M | 102 | intracerebral haemorrhage | P | Y |
| MCS4 | 37 | M | 67 | respiratory failure | P | Y |
| MCS5 | 47 | M | 49 | PICA infarct | P | Y |
| MCS6 | 47 | M | 52 | traumatic brain injury | S | Y |
| MCS7 | 34 | M | 51 | traumatic brain injury | S | Y |
| MCS8 | 54 | F | 74 | subarachnoidal haemorrhage | S | Y |
| MCS9 | 46 | M | 34 | multiple cerebrale infarcte | S | Y |
| MCS10 | 65 | M | 85 | intracerebral haemorrhage | S | Y |
| MCS11 | 31 | M | 66 | traumatic brain injury | S | Y |
| MCS12 | 52 | M | 146 | subarachnoidal haemorrhage | S | Y |
| MCS13 | 71 | F | 355 | subarachnoidal haemorrhage | S | N |
| MCS14 | 66 | F | 224 | intracerebral haemorrhage | S | Y |
| MCS15 | 53 | F | 100 | subarachnoidal haemorrhage | S | Y |
| MCS16 | 41 | F | 40 | cardiopulmonary resuscitation | S | Y |
| MCS17 | 71 | F | 70 | cardiopulmonary resuscitation | S | Y |
| MCS18 | 43 | M | 75 | traumatic brain injury | S | Y |
| MCS19 | 32 | F | 37 | encephalitis | S | Y |
| MCS20 | 33 | M | 91 | encephalitis | S | Y |
| MCS21 | 85 | M | 66 | subarachnoidal haemorrhage | S | Y |
| MCS22 | 18 | M | 937 | traumatic brain injury | S | N |
| Missing1 | 20 | M | missing | missing | S | N |
| Missing2 | 19 | M | missing | missing | S | N |
